# Cardiac surgery-associated acute kidney injury in a single healthcare system: a retrospective observational study

**DOI:** 10.62675/2965-2774.20260370

**Published:** 2026-06-02

**Authors:** Amit Prabhakar, Ceressa T. Ward, Ansley Morgan Tidwell, Isabel Won Angeles, David W. Boorman, Jun Ma, Vanessa Moll

**Affiliations:** 1 Emory University School of Medicine Department of Anesthesiology Atlanta Georgia United States Department of Anesthesiology, Emory University School of Medicine - Atlanta, Georgia, United States.; 2 Emory University Hospital Midtown Department of Pharmacy Atlanta Georgia United States Department of Pharmacy, Emory University Hospital Midtown - Atlanta, Georgia, United States.; 3 University of Minnesota Division of Critical Care Medicine Department of Anesthesiology Minneapolis Minnesota United States Department of Anesthesiology, Division of Critical Care Medicine, University of Minnesota - Minneapolis, Minnesota, United States.

**Keywords:** Acute kidney injury, Cardiac surgical procedures, Length of stay, Patient discharge, Renal replacement therapy

## Abstract

**Objective:**

To assess the incidence of cardiac surgery-associated acute kidney injury and to evaluate secondary outcomes, including 30-day mortality, prolonged mechanical ventilation, kidney replacement therapy, renal recovery, and length-of-stay metrics.

**Methods:**

This retrospective observational study analyzed routinely recorded data from a single multi-hospital healthcare system, including all adult cardiac surgery intensive care unit patients between July 1st, 2021, and June 30, 2022. Cardiac surgery-associated acute kidney injury was stratified according to the KDIGO guidelines using serum creatinine. Urine output criteria were incorporated when available.

**Results:**

Cardiac surgery-associated acute kidney injury of any stage occurred in 16.7% of patients (stage 1 - 13.6%, stage 2 - 1.8%, and stage 3 - 1.3%). Thirty-day mortality had wide confidence intervals, with odds ratios of 20.8 (95%CI 3.8 - 114) and 93.6 (95%CI 23.4 - 375) for acute kidney injury stages 2 and 3, respectively, compared with stage 0. A total of 27.5% (52/189) of patients with cardiac surgery-associated acute kidney injury did not meet the strict definition of renal recovery at discharge. Median intensive care unit length of stay, average time on mechanical, and time to discharge were significantly longer in patients with cardiac surgery-associated acute kidney injury (p < 0.001).

**Conclusion:**

Cardiac surgery-associated acute kidney injury is a common postoperative complication associated with substantially increased mortality, prolonged ventilation, longer length of stay, and reduced renal recovery. These findings underscore the clinical importance of early identification, risk stratification, and perioperative management strategies to mitigate harm from acute kidney injury.

## INTRODUCTION

Cardiac surgery-associated acute kidney injury (CSA-AKI) is a common postoperative complication affecting 5 - 42% of patients.^(1–3)^ The pathophysiology of CSA-AKI is complex and involves a combination of ischemia-reperfusion injury, inflammation, hemodynamic instability, and nephrotoxic exposures during the perioperative period.^(4,5)^ Cardiac surgery-associated acute kidney injury is associated with higher perioperative and long-term mortality, prolonged intensive care unit (ICU) and hospital length of stay (LOS), and increased healthcare costs.^(6–8)^ Patients who survive remain at risk of premature chronic kidney disease (CKD), even if renal function initially recovers.^(9)^ Up to 5% of patients subsequently require ongoing kidney replacement therapy (KRT).^(10,11)^

The Society of Thoracic Surgeons (STS) rating system incorporates the incidence of CSA-AKI,^(12)^ which will be a reportable Centers for Medicare and Medicaid Services (CMS) outcome measure (CMS832v1) in 2025.^(13)^ The Kidney Disease Improving Global Outcomes (KDIGO) criteria^(14)^ are used to classify AKI according to urine output and serum creatinine (SCr) in three stages of severity. The current STS definition of AKI, KDIGO stage 3 (defined as a 3-fold increase in SCr, a SCr > 4mg/dL, or the initiation of KRT), fails to identify the majority of patients with AKI. Recent studies have emphasized the prognostic value of mild to moderate AKI, which is associated with progressive morbidity and increased mortality.^(15–17)^

Patients with mild to moderate stage AKI (KDIGO stages 1 - 2) represent a critical group for targeted intervention. Prior studies evaluating the KDIGO bundle in high-risk cardiac surgery patients identified through biomarkers demonstrated a reduction in AKI progression when preventative strategies are initiated early.^(18–20)^ This underscores the clinical relevance of detecting even mild forms of CSA-AKI.

The objective of this study was to assess the incidence of CSA-AKI and to evaluate secondary outcomes, including 30-day mortality, prolonged mechanical ventilation, kidney replacement therapy, renal recovery, and LOS metrics.

## METHODS

### Study design and participants

A retrospective observational cohort study was conducted to evaluate the incidence of CSA-AKI. The Institutional Review Board (IRB) approved this minimal-risk study (Atlanta, Georgia, USA; IRB00004961), including a waiver for written Informed Consent. Utilizing the STS Adult Cardiac Surgery Database (ACSD) version 2.9,^(21)^ a report cross-referencing patients who underwent coronary artery bypass graft (CABG) and/or valve repair or replacement surgery during the specified timeframe of July 1st, 2021, to June 30, 2022, was generated. A manual review of the electronic medical record was conducted for patients 18 years of age or older admitted to a Cardiothoracic Surgery Intensive Care Unit (CTS ICU) for> 24 hours within this academic tertiary care health system for postoperative management after conventional CABG and/or valve repair or replacement.

Patients who underwent transcatheter aortic valve replacement, minimally invasive valve repair or replacement, robotic-assisted valve repair or replacement, robotic-assisted CABG, or reoperation within 72 hours of the initial procedure were excluded. Patients were also excluded if they were transferred from an outside hospital, had a history of CKD, end-stage renal disease (ESRD), renal transplant, or presented with AKI. Because excluding CKD patients may limit generalizability, we included them in a sensitivity analysis.

### Data collection

Using the institution's STS clinical data warehouse, data were extracted from the electronic medical records for all patients who met the inclusion criteria. The following demographic information was retrieved: age, sex, race/ethnicity, weight, admitting CTS ICU, history of diabetes mellitus, and STS score for predicted postoperative renal failure. Clinical data were obtained from the pre-, intra-, and post-operative periods. Preoperative data included baseline SCr and utilization of contrast media within 7 days prior to surgery. The intraoperative data included the use of cardiopulmonary bypass (CPB) or blood products, the duration of surgery, and extubation in the operating room. Postoperative kidney data included peak SCr, initiation and duration of KRT, use of KRT after discharge, rate of renal recovery, urine output within 7 postoperative days, and utilization of nephrotoxic medications (aminoglycoside, diuretics, vancomycin, and combination piperacillin-tazobactam plus vancomycin therapy) within 7 postoperative days. Additional postoperative variables included time on mechanical ventilation, ICU LOS, the incidence of new-onset atrial fibrillation and time to discharge. urine output data were missing for a substantial subset of patients and were therefore analyzed using available-case analysis; patients without recorded urine output could not be staged using urine output criteria. The present report was drafted in accordance with the Strengthening the Reporting of Observational Studies in Epidemiology (STROBE) statement for cohort studies.^(22)^

### Definitions

Acute kidney injury was defined per KDIGO criteria based on SCr;^(14)^ additional definitions are described in table 1.

**Table 1:** Terminology and definitions

Terminology	Definitions
AKI	Diagnosis and stage based on a predetermined rise in SCr, from baseline, within a specific time frame as outlined by the KDIGO^(14)^ guidelines
Baseline SCr	Based on laboratory data availability between 365 and 7 days prior to surgery. If SCr was unavailable, the highest SCr recorded at admission was used
Peak SCr	The highest SCr was within 7 postoperative days
Renal recovery	A decline in SCr to within 0.3mg/dL of baseline
Duration of AKI (days)	Time from AKI diagnosis to renal recovery

AKI - acute kidney injury; SCr - serum creatinine; KDIGO - Kidney Disease Improving Global Outcomes.

### Outcomes

The primary outcome of this study was the incidence of CTS patients who developed AKI (as defined by KDIGO) prior to ICU discharge. Secondary outcomes included 30-day mortality rate, prolonged mechanical ventilation (in hours), the initiation and duration of KRT (in days), ICU LOS (in days), hospital LOS (in days), and renal recovery at discharge.

### Power analysis

An *a priori* power analysis was not conducted. Instead, a sensitivity power analysis was conducted using G*Power 3.1.9.4.^(23)^ With a sample size of 1,130, a probability of 30-day mortality of 0.53% in patients without AKI, a proportion of patients in the AKI stage 1 group at 0.141 alpha threshold to reject the null hypothesis at 0.05 and a power of 80%, a two-tailed logistic regression test would be able to detect an odds ratio of 6.83 or greater.

### Statistical methodology

Data was analyzed using SAS 9.4 (Cary, NC). Categorical variables were analyzed with the Fisher exact test (2 x 2 tables) or the Chi-squared test (n x m tables). Continuous data were analyzed with the Mann-Whitney U test (equivalent to the t-test) or the Kruskal-Wallis test (equivalent to analysis of variance; Anova). *Post hoc* differences in the Kruskal-Wallis test were assessed using the Dwass-Steel-Critchlow-Fligner (DSCF) method. Differences in 30-day mortality were assessed by binary logistic regression. With two primary and four secondary outcomes, the risk of at least one false positive by random chance increases from the alpha threshold of 0.05 to 0.265. To mitigate this problem, the Hochberg method^(24)^ was employed, in which p values are placed in rank order and the alpha threshold for rejecting the null hypothesis is lowered stepwise: 0.05, 0.025, 0.167,… 0.0083.

### Multiple testing

Because of the large sample size, all resulting p-values were below the adjusted alpha threshold. Several continuous outcomes were significantly correlated, Spearman rank correlation, r = 0.16 - 0.57. In theory, they could have been analyzed simultaneously first using Multivariate Analysis of Covariance (MANCOVA). However, because p-values were already very low, this level of complexity was deemed unnecessary.

### Sensitivity analysis

We performed a sensitivity analysis by repeating the primary logistic regression after sequentially adding or excluding specific patient subgroups. Patients with CKD (n = 64) and those with a history of prior AKI (n = 5) were excluded from the primary analysis but were reintroduced in the sensitivity analysis. We also evaluated the effect of patients with reduced estimated glomerular filtration rate (eGFR) without prior CKD diagnosis by calculating eGFR using the Cockcroft–Gault equation, adjusted for body mass index, and applying a threshold of GFR < 60mL/minute to define reduced renal function. For each sensitivity run, the same logistic regression model of AKI stage *versus* 30-day mortality was rerun without altering preliminary tables. These analyses allowed us to assess whether including patients with diagnosed CKD, those with reduced eGFR without prior CKD diagnosis, or other exclusions materially influenced the direction or significance of the main findings.

## RESULTS

**Figure 1:**
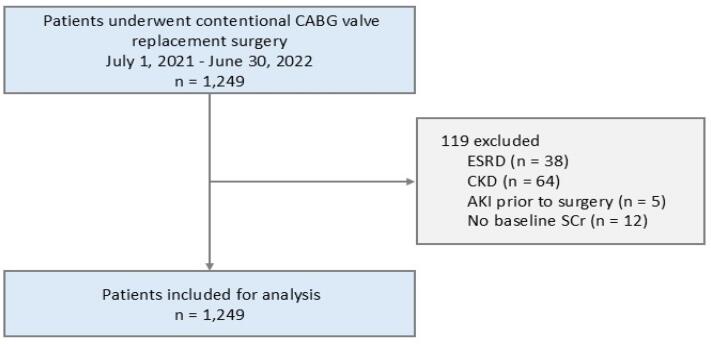
Inclusion/exclusion flow chart.

A total of 1,249 patients underwent cardiac surgery, of whom 1,130 were included in the final analysis (Figure 1). The surgeries performed include CABG (63%), valve repair/replacement surgery (32%) or both (5%). Demographic and perioperative clinical information is summarized in table 2. Patients who identified as Black or Hispanic were more likely to have AKI at any stage (26% *versus* 14%). They were also significantly more likely to have had a mitral valve replacement (15% *versus* 7%, p = 0.0002), to have diabetes (44% *versus* 34%, p = 0.0022), and to have a higher body mass index (median 29.4 *versus* 28.0, p = 0.0026), but had similar weight (p = 0.76). They were a median of 3 years younger (p = 0.0001) and had a higher median STS predicted renal failure (1.4% *versus* 0.76%; p < 0.0001). Non-Hispanic White patients were significantly older than patients of other races and ethnicities (p < 0.0001, Mann-Whitney U Test), with a median age (interquartile range [IQR]) of 66 (58 - 72) compared to 62 (54 - 70) for others.

CABG - coronary artery bypass surgery; ESRD - end-stage renal disease; CKD - chronic kidney disease; AKI - acute kidney injury; SCr - serum creatinine.

**Table 2:** Patient characteristics and demographics

Variable			AKI stage			p value*
			0	1	2 - 3	
Total			941	154	35	N/A
Demographics and medical history						
	Female		270 (29)	39 (25)	13 (37)	0.36
	Race					0.0002†
		White	636 (68)	81 (53)	22 (63)	
		Black	169 (18)	52 (34)	8 (23)	
		Asian	73 (8%)	11 (7)	0 (0)	
		Hispanic	43 (5)	9 (6)	3 (9)	
		Other/unknown	20 (2)	1 (1)	2 (6)	
	Hospital					
		ESJH	661 (70)	81 (53)	14 (40)	< 0.0001
		EUHM	193 (21)	45 (29)	12 (34)	
		EUH	87 (9)	28 (18)	9 (26)	
	Diabetes mellitus (yes)		325 (35)	73 (47)	17 (49)	0.0030
	Age (years)		64 [56 - 71]	68 [60 - 73]	62 [55 - 75]	0.0017†
	Weight (kg)		85 [73 - 98]	90 [75 - 104]	82 [72 - 105]	0.019†
	BMI (kg/m^2^)		28.0 [25 - 32]	29.8 [26 - 33]	29.4 [25 - 36]	0.0007†
	Serum creatinine					
		Baseline (mg/dL)	0.96 [0.8 - 1.1]	1.04 [0.9 - 1.2]	0.90 [0.8 - 1.3]	0.0018†
Surgical descriptions						
	New-onset atrial fibrillation		247 (26)	33 (21)	6 (17)	0.23
	Intraoperative blood products		158 (17)	46 (30)	13 (37)	< 0.0001
	CPB (yes)		758 (81)	133 (86)	30 (86)	0.18
	CPB time (minute)		120 [98 - 149]	122 [96 - 152]	123 [86 - 177]	0.96
	Surgical procedure					
		CABG	589 (63)	100 (65)	23 (68)	< 0.0001
		Mitral valve replacement	171 (18)	8 (5)	0 (0)	
		Aortic valve replacement	80 (8)	8 (5)	4 (12)	
		Other valve‡	58 (6)	25 (16)	5 (15)	
		CABG and valve	43 (5)	13 (8)	2 (6)	
Medical procedures and treatments						
	# of preoperative contrast studies					
		1	40 (4.2)	4 (2.6)	2 (5.7)	0.40†
		2 - 5	66 (7.0)	7 (4.6)	2 (5.7)	
	# of postoperative contrast studies					
		0 - 1	934 (99)	149 (97)	33 (94)	0.0004
		2 - 4	7 (1)	5 (3)	2 (6)	
	On ventilator (yes)		662 (70)	130 (84)	30 (86)	0.0003
	Extubation in the operating room (yes)		283 (30)	24 (16)	6 (17)	0.0004
	New KRT					
		No	N/A	154 (100)	26 (74)	< 0.0001§
		Yes	N/A	0 (0)	8 (26)	
	KRT after discharge					
		No	N/A	111 (100)	19 (83)	0.0040
		Yes	N/A	0 (0)	4 (17)	
	Vancomycin (yes)		51 (5.4)	21 (14)	14 (40)	< 0.0001
	Vancomycin, piperacillin & tazobactam (yes)		9 (1)	7 (4.6)	5 (14)	<0.0001
	Diuretics (yes)		887 (94)	152 (99)	34 (97)	0.016†
	Aminoglycosides (yes)		4 (0.4)	1 (0.7)	5 (14)	< 0.0001
	STS predicted renal failure (yes)		0.792 [0.49 - 1.4]	2.02 [1.0 - 3.3]	2.04 [1.0 - 3.1]	< 0.0001

AKI - acute kidney injury; ESJH - Emory St. Joseph's Hospital; EUH - Emory University Hospital; EUHM - Emory University Hospital Midtown; BMI - body mass index; CPB - cardiopulmonary bypass; CABG - coronary artery bypass surgery; KRT - kidney replacement therapy; STS - Society of Thoracic Surgeons.

* Chi-squared test for n x m categorical data; Fisher's exact test for 2 x 2 tables; Kruskal-Wallis test for continuous variables with acute kidney injury in three groups; Mann-Whitney U test for continuous variables with acute kidney injury no *versus* yes;

† comparing no acute kidney injury to acute kidney injury at any stage. For continuous variables, acute kidney injury stages 2-3 are not significantly different from stages 0 or 1 when divided into 3 groups;

‡ mitral valve replacement alone (n = 70) or with aortic valve replacement (n = 18);

§ all 8 patients with new kidney replacement therapy were acute kidney injury stage 3, *versus* n = 6/205 (2.9%) at acute kidney injury stage 3 in no new kidney replacement therapy, Fisher exact test. Results expressed as n, n (%) or median (interquartile range).

The presence of AKI at any stage was also significantly higher in patients with increased age, body weight, and baseline SCr, as well as diabetic patients and those admitted to Emory University Hospital (EUH). Patients who underwent mitral valve repair were least likely to develop AKI (4.5%), compared with those who underwent aortic valve replacement (13%), CABG (17%), or both (26%). Those who had mitral valve replacement, either alone or with another procedure, were most likely to develop AKI (34%). There were no significant differences between the groups with no AKI and AKI at any stage based on sex.

### Primary outcome: development of CSA-AKI

Cardiac surgery-associated acute kidney injury developed in 16.7% of patients (AKI stage 1 - 13.6%, stage 2 - 1.8%, and stage 3 - 1.3%).

Patients with AKI were more likely to have completed two or more postoperative contrast studies (p = 0.0039), to be on mechanical ventilation (p = 0.0003), to have received vancomycin with or without piperacillin-tazobactam (p < 0.0001), or to have received intraoperative blood products (p < 0.0001). The use of diuretics was significantly lower in patients without AKI (94%) than in AKI stages 1 and 2 - 3 combined (98%; p = 0.016). A significant difference in STS-predicted renal failure was observed between no AKI and any AKI; however, no significant difference was noted between AKI stage 1 and stage 2 - 3.

### Secondary outcomes

**Figure 2:**
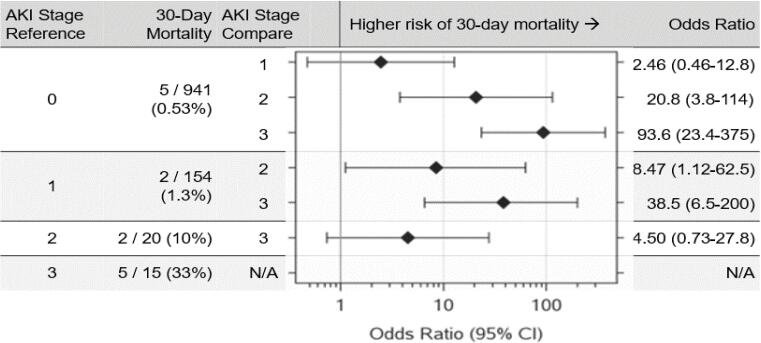
Forest plot risk of 30-day mortality.

**Figure 3:**
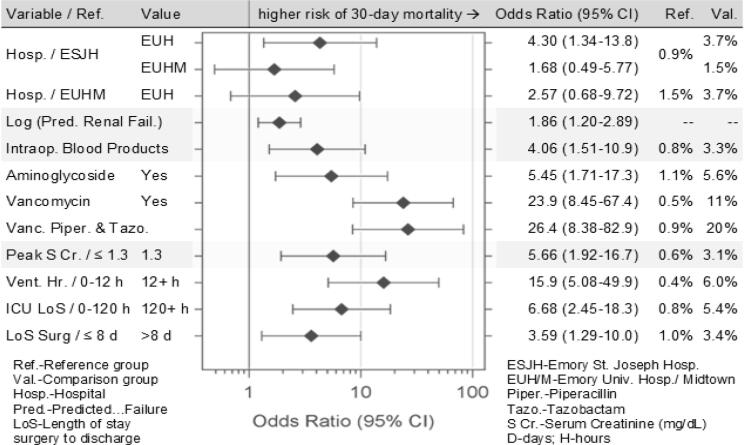
30-day mortality odds ratio for additional variables.

The incidence of 30-day mortality was significantly higher in patients with AKI stages 2 - 3. Compared to patients without AKI, the risk of 30-day mortality was 21 times higher in patients with AKI stage 2 and nearly 94 times higher in AKI stage 3 (Figure 2). The 30-day mortality was significantly higher at the largest of the three hospitals included (3.7%, odds ratio [OR] 3.67 [95% confidence interval; 95%CI] 1.26 - 10.7) compared to the other hospitals (1.0%). The odds ratio for this in an univariable logistic regression model was 3.67 (95%CI 1.26 - 10.7). STS score for predicted postoperative renal failure was also significantly higher, with an odds ratio of 1.86 (95%CI 1.20 - 2.89). A multivariable logistic regression model included age and AKI stage, with an odds ratio per decade of 1.97 (95%CI 1.14 - 3.40). The odds ratio for 30-day mortality with additional variables is shown in figure 3.

AKI - acute kidney injury; 95%CI - 95% confidence interval.

The figure above shows the 30-day mortality odds ratio for hospital, predicted renal failure, intraoperative blood products, intraoperative drugs, peak serum creatinine, and various surgical times. The percentages in the right two columns reflect the unadjusted 30-day mortality rates. Emory University Hospital's having a higher risk of mortality is likely an artifact of accepting sicker patients, as measured by a significantly higher predicted renal failure and a higher proportion of patients at acute kidney injury stages 1 - 3. Additional intraoperative and outcome variables were also checked but did not have a significantly higher risk of 30-day mortality. This includes new-onset atrial fibrillation, being on a ventilator, being extubated in the operating room, being on diuretics, procedure type, preoperative or postoperative contrast images, baseline serum creatinine, cardiopulmonary bypass time, time in the operating room, and the lowest urine output in the past 7 days. When ventilator hours were entered into the multivariable logistic regression model with age and acute kidney injury stage, the odds ratio for acute kidney injury stage 2 - 3 relative to acute kidney injury stage 0 or 1 was reduced by half to 18.1 (95%CI 5.3 - 62) and 17.9 (95%CI 3.4 - 91), respectively. Neither intraoperative blood products nor peak serum creatinine were included in the multivariable model.

Other secondary outcomes included prolonged mechanical ventilation (in hours), the initiation and duration of KRT (in days), ICU LOS (in days), and hospital LOS (in days). There was no significant difference in surgical time between patients with and without CSA-AKI; however, median ICU LOS, average time on mechanical ventilation, and time to discharge were significantly longer in patients with CSA-AKI (p < 0.001) (Table 3). During hospitalization, 0.71% of patients needed KRT. At discharge, renal recovery was not achieved in 27.5% (52/189) of patients, and 0.27% remained KRT-dependent.

**Table 3:** Outcomes by acute kidney injury stage

Variable			AKI stage*			p value*
			0	1	2 - 3	
Total			941	154	35	N/A
	30-day mortality (yes)		5 (0.53)	2 (1.3)	7 (20)	< 0.0001
	Minimum urine output in 7 days (mL/kg/hour)		No data	0.62 [0.42 - 0.85]	0.38 [0.28 - 0.50]	0.0002
	Peak serum creatinine (mg/dL)		1.01 [0.9 - 1.2]	1.52 [1.3 - 1.7]	2.70 [1.8 - 4.6]	< 0.0001†
	Renal recovery (yes)		N/A	124 (81)	13 (37)	< 0.0001
Length of stay						
	Time in operating room (hours)		6.6 [5.8 - 7.4]	7.0 [6.2 - 8.0]	6.5 [5.7 - 8.4]	0.0002†
	ICU (hours)		48 [28 - 71]	71 [46 - 99]	147 [51 - 311]	< 0.0001†
	Ventilator (hours)					
		Omits no time	5.0 [3.5 - 9.2]	8.2 [4.3 - 17.8]	16.3 [3.9 - 151]	< 0.0001
	Surgery-discharge (days)		4 [4 - 6]	6 [4 - 10]	13 [7 - 22]	< 0.0001
	AKI time (days)		N/A	1 [1 - 3]	3.5 [2 - 4.5]	0.016

AKI - acute kidney injury; ICU intensive care unit.

* Chi-squared test for n x m categorical data; Fisher exact test for 2 x 2 tables; Kruskal-Wallis test for continuous variables with 3 groups; Mann-Whitney U test for continuous variables with acute kidney injury no *versus* yes.

† peak serum creatinine: stage 0 < stage 1 < stage 2 - 3; time in operating room: stage 1 significantly greater than stage 0. Intensive care unit length of stay: stage 1 > stage 0 and stage 2 - 3 > stage 1 and 0.

### Sensitivity analysis

**Figure 4:**
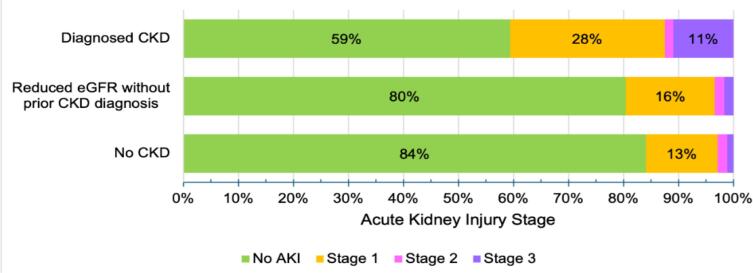
Distribution of acute kidney injury stages by baseline kidney function status.

Including patients with CKD (n = 64) and prior AKI (n = 5) in the analysis slightly attenuated the association between AKI stage and 30-day mortality but did not alter the overall conclusions. In the sensitivity model, CKD status itself did not enter the logistic regression (OR 2.61, 95%CI 0.58 - 11.7) and did not confound the relationship between AKI stage and mortality. Compared with the primary analysis, the odds ratios for AKI stage versus 30-day mortality were modestly reduced (e.g., AKI stage 3 *versus* stage 0: OR 64.9 [95%CI 18.7 - 224] *versus* 93.6 [95%CI 23.4 - 375]), yet remained statistically significant. Thirty-day mortality was 3.2% among diagnosed CKD patients (n = 2/64), compared with 1.2% in those without CKD (n = 11/903) and 1.3% in patients with reduced eGFR without prior CKD diagnosis (n = 3/229); differences were not statistically significant (Fisher's exact test, p = 0.21). Additionally, AKI stage distribution differed significantly between patients with diagnosed CKD and those without (p < 0.0001) (Figure 4), but not between those with reduced eGFR without prior CKD diagnosis and patients without CKD (p = 0.40).

CKD - chronic kidney disease; AKI - acute kidney injury.

## DISCUSSION

In this retrospective analysis within a multi-hospital academic system, we found that the severity of CSA-AKI was a major determinant of 30-day mortality, with patients experiencing stage 2 - 3 AKI exhibiting the greatest risk. The development of postoperative AKI (all stages) was observed in 16.7% of patients, with the majority classified as KDIGO stage 1. Compared with stage 0 (no AKI), patients with CSA-AKI had significantly longer mechanical ventilation duration, ICU LOS, and hospital LOS. Although prior studies have reported increased mortality among patients with stage 1 AKI, we did not observe this association. This likely reflects heterogeneity in patient risk profiles across cardiac surgery populations. In our cohort, patients with stage 1 AKI tended to be younger with fewer comorbid conditions and experienced higher rates of renal recovery, which may have attenuated short-term mortality risk. The risk in patients with stages 2-3 was up to 94 times higher than in patients without AKI. Poor renal recovery in a significant subset of patients was also observed; notably, 27.5% of patients with AKI failed to return to baseline kidney function by the time of discharge. This aligns with prior evidence showing that incomplete renal recovery after AKI substantially increases long-term risk of CKD, with up to one-quarter of patients progressing to CKD within 3-5 years.^(25,26)^ Our findings emphasize that CSA-AKI is a frequent and serious postoperative complication with long-term impact and an increased risk of mortality. Because 30-day mortality is a widely used quality and outcome benchmark in cardiac surgery, these results underscore the importance of preventing, identifying, and following patients with suboptimal recovery.

The sensitivity analyses further support the robustness of our findings. When patients with diagnosed CKD and prior AKI were included, the strength of the association between AKI stage and 30-day mortality was modestly attenuated but remained highly significant. Consistent with prior literature, patients with CKD demonstrated a higher incidence of AKI at all stages compared to those without CKD, underscoring their increased perioperative vulnerability. Importantly, CKD status itself did not independently predict 30-day mortality, nor did it confound the relationship between AKI stage and outcomes. Similarly, when evaluating patients with reduced eGFR without prior CKD diagnosis, mortality rates and AKI stage distribution did not differ significantly from those without CKD. Collectively, these findings indicate that while underlying kidney dysfunction influences AKI susceptibility, the severity of perioperative AKI remains the dominant driver of short-term mortality across patient subsets. These sensitivity analyses strengthen real-world clinical applicability by demonstrating that AKI staging retains its prognostic value even among patients with preexisting renal impairment, who constitute a large proportion of those undergoing cardiac surgery.

When comparing our findings to those of other studies, our observed incidence of AKI aligns with systematic reviews such as that of Hu et al.,^(27)^ which identified pooled incidence rates of 22.3% and 13.6%, 3.8%, and 2.7% at stages 1, 2, and 3, respectively. The slightly lower incidence in our study may be attributable to differences in surgical practices, patient demographics, or the exclusion of patients with pre-existing renal conditions. Our observed CSA-AKI incidence also highlights the practical reality that kidney injury remains a common and clinically meaningful postoperative complication and supports the need for routine perioperative risk stratification and the implementation of evidence-based AKI prevention bundles. Approximately 3% of patients in this study required at least temporary KRT, which is comparable to the literature.^(10,28)^ Furthermore, our results affirm prior conclusions that severe AKI (KDIGO stage 2 or 3) substantially increases the risk of postoperative mortality and leads to prolonged ICU and hospital LOS. Machado et al.^(11)^ similarly found that patients with stage 3 AKI had the highest mortality risk, a trend mirrored in our study, where stage 3 AKI was associated with nearly a 94-fold increase in mortality. While this study did not include long-term outcomes, others have found that 25% of patients with AKI will suffer from CKD after 3 years.^(26)^

The association of CSA-AKI with extended mechanical ventilation, increased use of nephrotoxic medications, and intraoperative blood transfusions also aligns with findings from other research. The multifactorial nature of AKI development is well described, linking it to factors such as CPB, systemic inflammation, and ischemia-reperfusion injury.^(6,29)^ Our study similarly identified a higher incidence of AKI in patients requiring longer mechanical ventilation or receiving blood products, which further underscores the complexity of managing patients post-cardiac surgery. Racial and ethnic disparities in AKI incidence were also present in our cohort, consistent with previously published data demonstrating disproportionate AKI burden among Black and Hispanic patients. These disparities may reflect differences in baseline comorbidities, access to care, perioperative risk exposure, and broader social determinants of health, underscoring the need for targeted future research and interventions.

Our study has several strengths, including its large sample size and use of a multi-hospital academic dataset. We employed the KDIGO criteria for standardized AKI classification, enabling comparability with other studies and enhancing the reliability of our findings. Additionally, using the STS database enabled inclusion of detailed perioperative variables, thereby contributing to a comprehensive analysis of patient outcomes. However, there are notable limitations. The retrospective design limits our ability to establish causal relationships, and the lack of long-term follow-up precludes an assessment of the progression from AKI to CKD, a significant concern in patients who do not achieve full renal recovery. Although the sample size was large, the 30-day mortality rate for AKI stages 0 and 1 was low, and the sample sizes for AKI stages 2 and 3 were small, limiting the ability to assess differences between these stages due to lower statistical power. Urine output data were missing for a subset of patients, limiting the full application of KDIGO urine output criteria and potentially underestimating the true incidence and severity of CSA-AKI, as AKI classification relied primarily on SCr.

Furthermore, perioperative and postoperative hemodynamic variables were not abstracted, precluding adjustment for contributors to kidney injury. Moreover, our study did not assess the role of biomarkers in predicting AKI or guiding early intervention, which has been a focus of recent research. Multiple studies have demonstrated that implementing the KDIGO guidelines for high-risk patients identified through biomarkers can significantly reduce the incidence of CSA-AKI.^(18–20,30)^ Future studies could benefit from incorporating such biomarker-based approaches to identify patients at risk earlier and initiate preventative strategies.

## CONCLUSION

Our findings highlight that cardiac surgery-associated acute kidney injury remains a powerful predictor of short-term morbidity and mortality in contemporary cardiac surgery practice. Cardiac surgery-associated acute kidney injury increases mortality, prolongs hospital stays, increases cost, and can subsequently lead to incomplete renal recovery. While our study provides valuable insight into the incidence and outcomes of cardiac surgery-associated acute kidney injury in a multi-hospital academic system, further prospective research is needed to examine long-term renal outcomes and to evaluate the impact of early interventions, such as biomarker-guided therapies, on acute kidney injury incidence and severity. Integration of standardized KDIGO care bundles enhanced postoperative renal surveillance, and evidence-based preventative strategies may meaningfully improve outcomes for high-risk patients. By demonstrating the strong prognostic significance of acute kidney injury severity across diverse patient subgroups, this study underscores the critical need for broader efforts to reduce the expansive burden of cardiac surgery-associated acute kidney injury.

## Data Availability

Deidentified individual participant data underlying the results reported in this article will be made available from the corresponding author upon reasonable request after publication, contingent on approval by the Emory University IRB and execution of any required data use or sharing agreements. A detailed data dictionary and codebook will be provided, but data will not be posted in a public repository due to human subjects privacy considerations and institutional policies.
